# A Bayesian approach to estimate changes in condom use from limited human immunodeficiency virus prevalence data

**DOI:** 10.1111/rssc.12116

**Published:** 2015-08-10

**Authors:** J. Dureau, K. Kalogeropoulos, P. Vickerman, M. Pickles, M.‐C. Boily

**Affiliations:** ^1^ London School of Economics and Political ScienceUK; ^2^ London School of Hygiene and Tropical MedicineUK; ^3^ Imperial CollegeLondonUK

**Keywords:** Bayesian inference, Condom use, Epidemic modelling, Human immunodeficiency virus infections, Particle Markov chain Monte Carlo methods, Time varying parameter

## Abstract

Evaluation of large‐scale intervention programmes against human immunodeficiency virus (HIV) is becoming increasingly important, but impact estimates frequently hinge on knowledge of changes in behaviour such as the frequency of condom use over time, or other self‐reported behaviour changes, for which we generally have limited or potentially biased data. We employ a Bayesian inference methodology that incorporates an HIV transmission dynamics model to estimate condom use time trends from HIV prevalence data. Estimation is implemented via particle Markov chain Monte Carlo methods, applied for the first time in this context. The preliminary choice of the formulation for the time varying parameter reflecting the proportion of condom use is critical in the context studied, because of the very limited amount of condom use and HIV data available. We consider various novel formulations to explore the trajectory of condom use over time, based on diffusion‐driven trajectories and smooth sigmoid curves. Numerical simulations indicate that informative results can be obtained regarding the amplitude of the increase in condom use during an intervention, with good levels of sensitivity and specificity performance in effectively detecting changes. The application of this method to a real life problem demonstrates how it can help in evaluating HIV interventions based on a small number of prevalence estimates, and it opens the way to similar applications in different contexts.

## 1 Introduction

Significant resources are being committed to implement large‐scale interventions against infectious diseases such as the human immunodeficiency virus (HIV), that killed an estimated 2 million individuals in 2008 (UNAIDS and World Health Organization, 2009). Increasing attention is given to the evaluation of these large‐scale intervention programmes to understand what still needs to be done to control the epidemic and eventually to achieve elimination.

Even if antiretroviral therapy has become an important component of large‐scale prevention interventions, the use of condoms and circumcision remain important strategies for reducing HIV transmission. Although there are difficulties in estimating condom use trends accurately, due to biases that are inherent in self‐reported behaviour (Turner and Miller, 1997; Zenilman *et al*., 1995; Hanck *et al*., 2008), its average level closely determines the spread of HIV (Boily *et al*., 2007). Thus, it is important to assess whether trends in epidemiological data such as HIV prevalence can be used to infer the effect of interventions on risk behaviours that are susceptible to self‐reported bias. This is furthermore motivated by the fact that directly observed quantities such as HIV prevalence do not provide straightforward indications of the effect of an intervention. Indeed, even if an efficient intervention is introduced early, an epidemic has an intrinsic dynamic that can still lead to a growth in prevalence. Alternatively, in a mature epidemic the prevalence can decrease even though on‐going interventions are inefficient (Boily *et al*., 2002). Hence, it is the trajectory of the underlying condom use over time, and especially since the beginning of a prevention programme, that can shed light on the effect of the intervention and on the future trajectory of the epidemic. In this perspective, we develop a Bayesian approach to evaluate the Avahan programme, which is a large‐scale HIV and acquired immune deficiency syndrome intervention targeted to high‐risk groups, through the estimation of the evolution of condom use from the available HIV prevalence data.

The Avahan intervention was motivated by high levels of HIV prevalence among high‐risk groups observed in southern India (typically greater than 20%) (Ramesh *et al*., 2008), which lead to concerns about infections bridging to their long‐term partners and the general population. The programme was launched by the Bill and Melinda Gates Foundation in 2003 (Bill and Melinda Gates Foundation, 2008) and has targeted high‐risk groups for HIV infection, in particular female sex workers (FSWs), by promoting and distributing free condoms. Several studies have been conducted to examine the effect of the Avahan programme (Boily *et al*., 2008, 2007, 2013; Deering *et al*., 2008; Lowndes *et al*., 2010; Pickles *et al*., 2010, 2013), and to learn from it to inform future large‐scale interventions. A key part of such evaluations is examining how risk behaviours, chiefly the use of condoms, defined as the proportion of sex acts protected by condoms at a given time, have changed over the course of the intervention. However, this can be difficult to measure in practice. Baseline condom use may be difficult to record when an intervention needs to be implemented rapidly, as happened with the Avahan programme, or may be recorded only on few occasions. Although those targeted by the intervention may be asked about their condom use history (Lowndes *et al*., 2010), their answers may be subject to social desirability and recall biases. In principle, the total number of condoms sold or distributed can be enumerated (Bradley *et al*., 2010), but accurate records may not be available, condoms may be used for family planning by lower‐risk individuals and the distribution of condoms is not a guarantee of their correct usage (Bradley *et al*., 2010; Kumar *et al*., 2011). Thus, in addition to direct approaches through quantitative behavioural surveys or records of condom availability, model‐based methods can be used to infer unobserved quantities of interest, such as the use of condoms, and to complete the partial information that is available from observed quantities such as HIV prevalence, using knowledge of the dynamics of large‐scale epidemics.

A first study in the context of the Avahan programme was presented in Pickles *et al*. (2010, 2013) and Boily *et al*. (2013). In this work, a deterministic dynamic model for HIV or sexually transmitted infection was formulated on the basis of a compartmental representation incorporating heterogeneous sexual behaviour. The main aim of this approach was to shed light on the partially observed trajectory of condom use on the basis of different sources of data, with their own potential biases. This initial inference proceeded by setting three different hypothesized scenarios for the condom use evolution over time, before and after the introduction of the Avahan programme in 2003, that were estimated by using either self‐reported condom use data from a number of serial cross‐sectional surveys on FSWs (Lowndes *et al*., 2010) or using condom distribution and condom sales data. The three hypotheses alternatively made the following assumptions.

- The use of condoms initially increased according to the reported FSWs survey data until the Avahan initiative started in 2003 and was assumed to remain constant afterwards, despite evidence of an increase from FSW survey data. This was to acknowledge that FSWs may overreport the use of condoms after being exposed to the intervention.
- The use of condoms increased as in scenario (a) up to the Avahan initiative, but use was assumed to increase from 2003 onwards in accordance with self‐reported FSWs survey data.
- Scenario (c) is the same as scenario (b) but the increase after 2003 was in accordance with the condom distribution and sales data.

The HIV–sexually transmitted infection model was then used with each of these scenarios, which were therefore assessed on the basis of the fit to the available prevalence observations.

The work that we present in this paper operates in a similar context to that in Pickles *et al*. (2010) but, rather than making explicit assumptions about the evolution of condom use based on additional sources, we aim to develop a Bayesian inference framework exploring the entire space of the condom use trajectories without any reference to the 2003 Avahan intervention, FSWs surveys or condom distribution data. By assessing the effect of the Avahan intervention on condom use under less stringent assumptions, this approach provides additional and stronger evidence of the effect of the Avahan programme on condom use, beyond the three scenarios above. From a methodology perspective, the model formulation can be put in a state space setting where an underlying latent process (condom use trajectory) is observed through the prevalence data, and the link between these quantities is given by the deterministic model for the HIV infections. Inference in this context is a challenging task given the limited amount of HIV prevalence data aside from initial conditions (four observations) that are concentrated over a period of 6 years and are utilized to estimate a 25‐years‐long trajectory. Various models for the condom use trajectories were considered, including smooth and non‐differentiable (yet continuous) choices. In the remainder of this paper, the term ‘trajectory prior’ is used to refer to these models to avoid confusion with the deterministic HIV model. We also present a general and efficient computational scheme using Markov chain Monte Carlo (MCMC) techniques based on the particle MCMC (PMCMC) algorithm (Andrieu *et al*. (2010); see Dureau *et al*. (2013) for an application in a different context). The focus is on estimating the amplitude of the change in the use of condoms since 2003 (the start of the Avahan programme) to assess the effect of the Avahan intervention on condom use. In addition to the scientific interest of this application, there are various methodological challenges given the limited amount of data. For this reason, the properties of the estimators arising from the MCMC method are studied via simulations, and the performance is assessed from a decision‐making perspective through their sensitivity and specificity in detecting changes in the use of condoms.

The next section presents the models that are introduced in this paper, the data that are typically available for such studies and the way in which prior information is incorporated. The computational techniques, mainly the PMCMC algorithm, are also presented. The methodology that is employed to compare the performance of the proposed trajectory priors is presented in Section 3, and the results from this study are introduced in Section 4 along with an application to real data from the Indian acquired immune deficiency syndrome initiative Avahan. Finally, Section 5 concludes with some relevant discussion.

## 2 Models and methods

Our model specification consists of two parts. The first part is based on the formulation of Pickles *et al*. (2010) for the HIV transmission population dynamics. Our proposed methodology targets mainly the second part, where the HIV transmission model adopted is extended by considering a parametric time varying version of the condom use parameter. In what follows we first introduce and describe the HIV transmission model (Section 2.1), before getting into our proposed extension of it (Section 2.2), its prior parameterization (Section 2.3) and its MCMC implementation (Section 2.4).

### 2.1 Human immunodeficiency virus transmission model for female sex workers

**Figure 1:**
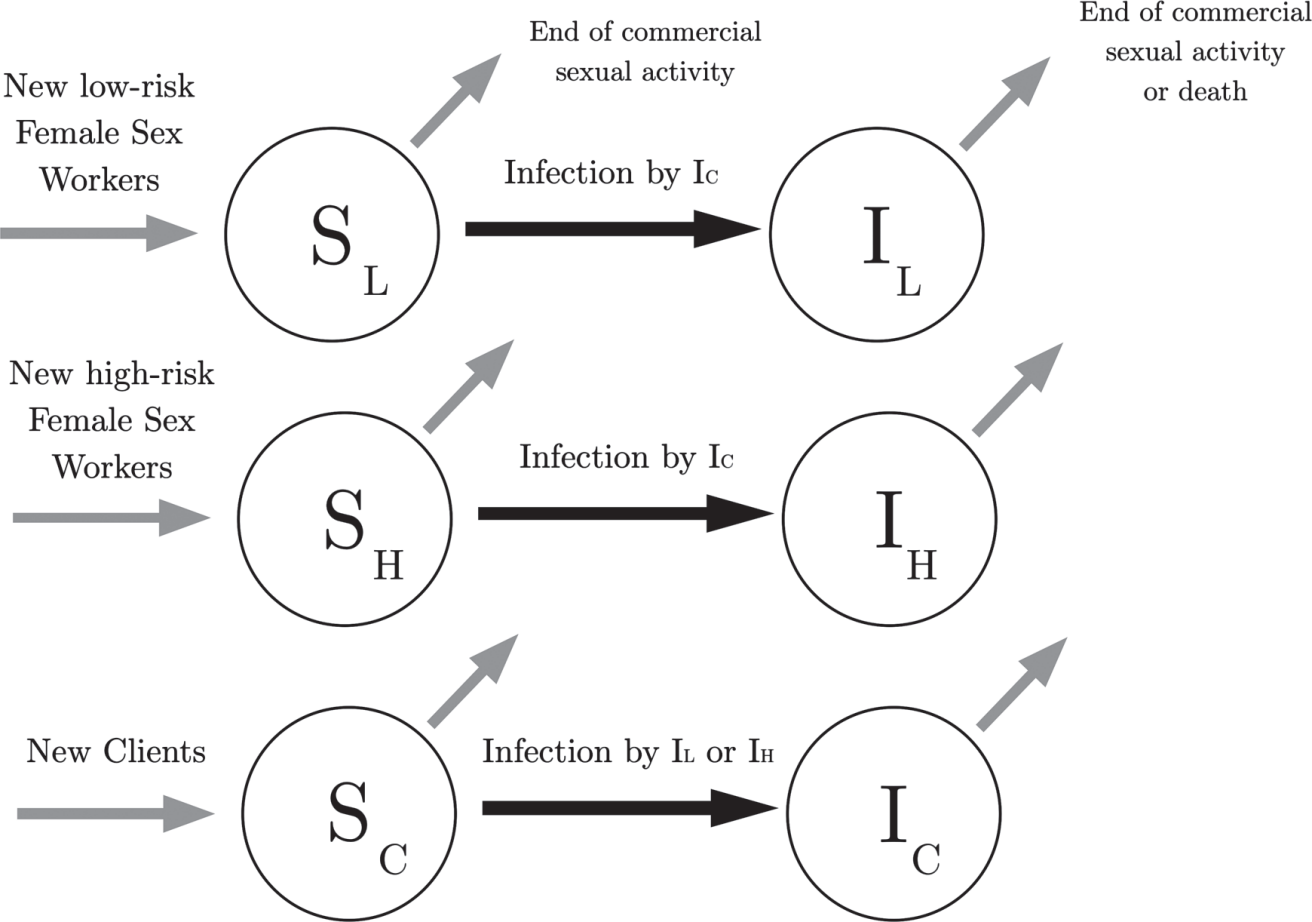
Flow diagram of the model

In this section, we present the part of our modelling framework that aims to capture the dynamics of HIV transmission in an environment such as the Mysore district, in southern India. The model is based on that of Pickles *et al*. (2010), focusing only on HIV transmission between FSWs and clients. Importantly, the model retained the essential heterogeneity in sexual risk behaviour that is observed in the data by stratifying FSWs in high and low volume (numbers of clients seen in a year) sex workers. A stable but open population is considered, consisting of FSWs and their clients. In line with Vickerman *et al*. (2010), we assume that the remaining members of the population (e.g. companions of clients or sex workers), who are not involved directly in sex work, have little influence on the dynamics of the epidemic and can therefore be ignored. The model structure groups FSW into high‐risk, *H*, and low‐risk, *L*, sex workers, who may have different numbers of clients, *C*. Each individual in these three groups can either be susceptible, *S*, to HIV infection, infectious, *I*, or retired either because of death or ceasing commercial sexual activity. Moreover, individuals who either decease or stop being involved in commercial sex are assumed to be replaced by susceptible individuals, such that the size of the risk population remains constant. A graphical representation of the model is given in Fig. 1 via a flow diagram for each of these three population groups. A key component of the epidemic model is the force of infection *β*(*t*) which is assumed to be the product between the number of clients and the probability of HIV transmission in at least one out of *a* sexual acts per client encounter. We denote respectively by *U*(*t*) and *e* the proportion of FSWs’ sex acts that are protected by condoms and the efficacy of condoms in protecting against transmission of HIV per sex act. A sexual act can lead to HIV transmission when either no condom is being used, occurring with probability 1−*U*(*t*), or when a condom is not being used effectively, occurring with probability *U*(*t*)(1−*e*). Hence, the combined probability of a sexual act that can lead to HIV transmission, which is also referred to as a risky sexual act, simplifies to 1−*eU*(*t*). Consequently, the respective forces of infection of high‐risk FSWs, low‐risk FSWs and clients can be defined as

(1) $$\left.\begin{array}{cc} & \beta_{H}\left(t\right)=C_{H}\left(1-{\left[1-p_{S}\left\{1-eU\left(t\right)\right\}\right]}^{a}\right),\\ & \beta_{L}\left(t\right)=C_{L}\left(1-{\left[1-p_{S}\left\{1-eU\left(t\right)\right\}\right]}^{a}\right),\\ & \beta_{C}\left(t\right)=\frac{C_{H}+C_{L}}{2}\frac{N_{L}+N_{H}}{N_{C}}\left(1-{\left[1-p_{C}\left\{1-eU\left(t\right)\right\}\right]}^{a}\right), \end{array}\right\}$$

where

- $C_{H}$ and $C_{L}$ are the numbers of clients of FSWs per month, which differ for high‐risk and low‐risk FSWs and
- $N_{C}$, $N_{L}$ or $N_{H}$ are the total numbers of clients, low‐risk and high‐risk sex workers respectively, considered to be constant over time. By construction, $N_{L}$ and $N_{H}$ are equal.
- $p_{S}$ or $p_{C}$ are the probability of HIV transmission from client to sex worker or sex worker to client respectively during an unprotected sex act.

Additionally, the transmission dynamics of HIV will depend on the following lengths of time.

- $\mu_{S}^{-1}$ or $\mu_{C}^{-1}$ are the average lengths of sexual activity as a sex worker or client respectively.
- $\alpha^{-1}$ is the average life expectancy with HIV.

Information on these quantities is available from various serial cross‐sectional biobehavioural surveys conducted in the Mysore district during the intervention (Ramesh *et al*., 2008). However, there is still uncertainty around these biological and behavioural parameters, which is reflected in the estimates of condom use by using a Bayesian approach (De Angelis *et al*., 2008).

The complete transmission dynamics model can be defined via the following set of differential equations:

(2) $$\left.\begin{array}{cc} & \frac{dS_{H}}{dt}\left(t\right)=-\beta_{H}\left(t\right)S_{H}\left(t\right)\frac{I_{C}\left(t\right)}{N_{C}}+\left(\mu_{S}+\alpha \right)I_{H}\left(t\right),\\ & \frac{dI_{H}}{dt}\left(t\right)=\beta_{H}\left(t\right)S_{H}\left(t\right)\frac{I_{C}\left(t\right)}{N_{C}}-\left(\mu_{S}+\alpha \right)I_{H}\left(t\right),\\ & \frac{dS_{L}}{dt}\left(t\right)=-\beta_{L}\left(t\right)S_{L}\left(t\right)\frac{I_{C}\left(t\right)}{N_{C}}+\left(\mu_{S}+\alpha \right)I_{L}\left(t\right),\\ & \frac{dI_{L}}{dt}\left(t\right)=\beta_{L}\left(t\right)S_{L}\left(t\right)\frac{I_{C}\left(t\right)}{N_{C}}-\left(\mu_{S}+\alpha \right)I_{L}\left(t\right),\\ \frac{dS_{C}}{dt}\left(t\right) & =-\beta_{C}\left(t\right)S_{C}\left(t\right)\{\frac{C_{H}N_{H}}{C_{H}N_{H}+C_{L}N_{L}}\frac{I_{H}\left(t\right)}{N_{H}}+\frac{C_{L}N_{L}}{C_{H}N_{H}+C_{L}N_{L}}\frac{I_{L}\left(t\right)}{N_{C}}\}+\left(\mu_{C}+\alpha \right)I_{C}\left(t\right),\\ & \frac{dI_{C}}{dt}\left(t\right)=\beta_{C}\left(t\right)S_{C}\left(t\right)\{\frac{C_{H}N_{H}}{C_{H}N_{H}+C_{L}N_{L}}\frac{I_{H}\left(t\right)}{N_{H}}\frac{C_{L}N_{L}}{C_{H}N_{H}+C_{L}N_{L}}\frac{I_{L}\left(t\right)}{N_{L}}\}-\left(\mu_{C}+\alpha \right)I_{C}\left(t\right). \end{array}\right\}$$

In these equations, the state of the epidemic over time is described by the number of susceptible individuals among clients $S_{C}$, among the low‐risk FSWs, $S_{L}$, and among the high‐risk FSWs, $S_{H}$, as well as the corresponding numbers of infected individuals, $I_{C}$, $I_{H}$ and $I_{L}$. Three types of constant parameters are involved: the initial prevalence among the various groups of interest at time $t_{0}=\text{January}\;1\text{st},1985$ ($\theta_{t_{0}}=\left\{S_{H}\left(t_{0}\right),I_{H}\left(t_{0}\right),S_{L}\left(t_{0}\right),I_{L}\left(t_{0}\right),S_{C}\left(t_{0}\right),I_{C}\left(t_{0}\right)\right\}$), time invariant parameters describing the biological and behavioural determinants of HIV transmission ($\theta_{b}=\left\{\mu_{S},\mu_{C},\alpha ,C_{H},C_{L},p_{S},p_{C},a,e\right\}$) and the parameters that play a role in the trajectory priors for condom use between 1985 and 2010 ($\theta_{c}$). All three components $\theta_{t_{0}},\theta_{b}$ and $\theta_{c}$ are integrated into a global vector of constant parameters, which is denoted by *θ*. The trajectory $X_{0:N}$ of the space vector $X\left(t\right)=\{S_{H}\left(t\right),I_{H}\left(t\right),S_{L}\left(t\right),I_{L}\left(t\right),S_{C}\left(t\right),I_{C}\left(t\right)\}$ at times $\left\{t_{0},\ldots ,t_{N}\right\}$ is then defined as a deterministic function of *θ* and *U* ($X_{0:N}=f\left(\theta ,U\right)$) through the HIV transmission model, and the resulting HIV prevalence points are compared with the available observations $y_{t_{1}},y_{t_{2}},\ldots ,y_{t_{N}}$, denoted by $y_{1:N}$. The function *f*(·) is not available in closed form but can be obtained given the trajectory of *U* by solving the above ordinary differential equations. More specifically, we introduce a time discretized version of *U*, with equidistant points in time of step *δ*, $U_{0:N}^{d}=\left\{U\left(t_{0}\right),U\left(t_{0}+\delta \right),U\left(t_{0}+2\delta \right),\ldots ,U\left(t_{N}\right)\right\}$. The partition of the condom use trajectory can be made arbitrarily fine through the user‐specified parameter *δ* to limit the approximation error that is induced by the time discretization. For ease of notation, the superscript *d* will be omitted in the remainder of the paper.

Assigning a model for the observation error provides the likelihood of the observation $y_{1:N}$ conditional on the *U*‐trajectory $p(y_{1:N}|\theta ,U)$. The data consist of HIV prevalence estimates obtained from random samples of FSWs and clients in the Mysore district obtained at different time points. The binomial distribution is parameterized at each $t_{i}$ by $\left\{I_{H}\left(t_{i}\right)+I_{L}\left(t_{i}\right)\right\}/\left(N_{H}+N_{L}\right)$ for prevalence observations among FSWs, and by $I_{C}\left(t_{i}\right)/N_{C}$ for prevalence observations among clients.

Overall, the model appearing in Fig. 1 and equations (1) and (2) contains fewer parameters than the model in Pickles *et al*. (2010). This was done mainly for parsimony; models of increased complexity can be used, provided that there is adequate prior information on their parameters. More details on informative priors are provided in Section 2.3

### 2.2 Trajectory priors for condom use

From a modelling perspective, the main contribution of this paper lies in the introduction of various models for the evolution of the use of condoms, aiming to explore the complete space of trajectories rather than restricting the analysis to a limited set of scenarios. First, we assign a trajectory prior aiming to impose little constraint on the trajectory of condom use over time. We proceed by assigning Brownian motion to a transformed version of *U* such that it takes values on the real line. An alternative similar formulation could use integrals of Brownian motion. Loosely speaking, these models can be linked with a smoothing splines approach (Wahba, 1990). The second objective aims at a slightly more informative formulation regarding the evolution of condom use and considers sigmoid‐shaped growth curves. Such growth curves, which were also mentioned in Pickles *et al*. (2010), provide natural models for related quantities and have been used in various contexts such as biology (Zwietering *et al*., 1990), marketing (Lessne and Hanumura, 1998) and epidemiology (Omran, 1971). Hence, the second trajectory prior is based on the generalized deterministic Bertalanffy–Richards (DBR) model (see for example Garcia (1983) and Yuancai *et al*. (1997)). To enrich this context and to address estimation issues that can be encountered with the DBR model (Lei and Zhang, 2004), we also consider an alternative empirical sigmoid curve model. In what follows, we denote with *x* the latent process that drives the condom use trajectory, which in turn provides the link with the prevalence observations through the model in Section 2.1.

#### 2.2.1 Brownian motion

The first formulation assigns Brownian motion to a transformed version of the condom use trajectory. As the latter must be constrained in the [0,1] region, we work with the logit transformation of *U*(*t*), which is denoted by

(3) $$\begin{array}{cc} U(t)= & \frac{\exp\{x(t)\}}{1+\exp\{x(t)\}},\\ & dx\left(t\right)=\sigma dB_{t}. \end{array}$$

The use of diffusion processes to describe time varying quantities in contexts that are associated with uncertainty has been explored in the context of epidemiology; see for example Cazelles and Chau (1997), Cori *et al*. (2009) and Dureau *et al*. (2013). It can also be seen as a prior according to which *x*(*t*) is a random walk with continuous, yet non‐differentiable, trajectories. It is used here in an attempt to incorporate a limited amount of prior information on the shape of the trajectory. It can also be used as an exploration tool for potential modelling–remodelling steps towards more informative formulations. In fact, inspection of the output from this model motivated the choice of sigmoid‐shaped growth curves, which are described in the next section. Variations of this formulation may include smoother diffusion models, by taking integrals of the Brownian motion, or alternative transformations such as the probit link. We note at this point that very little information is available on the volatility in model (3), which is determined mostly by its prior. More details are provided in Sections 2.3 and 3.

#### 2.2.2 Deterministic Bertallanfy–Richards function

Qualitatively, condom use trends reconstructions by alternative methods (Lowndes *et al*., 2010; Bradley *et al*., 2010) suggest that condom use was quite low in 1985 and has grown over recent years. The above motivated the use of a growth curve parametric model as an alternative to the Brownian motion diffusion model. We use the generalized Bertalanffy–Richards family (Richards, 1959; Garcia, 1983) that can be written as

$$U\left(t\right)=\eta {\left\{1-B\exp\left(-kt\right)\right\}}^{1/\left(1-m\right)},$$

or alternatively, in a differential equation framework,

(4) $$U\left(t\right)={\left\{\left(1-m\right)x\left(t\right)+\eta^{1-m}\right\}}^{1/\left(1-m\right)},$$

(5) dx(t)=−kx(t)dt.

The growth curve can be parameterized by four quantities: the initial value of $U_{0}$, the time of inflection $\tilde{t}$ (at which $U’(\tilde{t})=0$), the value of condom use after an infinite time *η* (also termed the asymptote) and the ‘shape’ or ‘allometric’ parameter *m*. The time of inflection can be related to the parameter *k* by

$$k\tilde{t}=\log\left(\frac{B}{1-m}\right).$$

This family contains various growth curves, including the logistic (*m*=2) and Gompertz (*m*→∞) functions. Furthermore, this definition implies that the initial value $U_{0}$ is lower than $m^{1/\left(1-m\right)\eta }$. To focus on sigmoid‐shaped growth curves, we restrict our attention to cases where *m*⩾1 (Yuancai *et al*., 1987).

#### 2.2.3 Deterministic empirical sigmoid curve

An empirical sigmoid model is also considered, to address the potential difficulties that can arise with the parameterization of the DBR model. First, inference on the allometric parameter *m* in the DBR has been shown to be problematic (Lei and Zhang, 2004). In addition, since growth models are used to study intrinsically growing objects, trajectories that are inexplicably stable for a long period of time and that eventually start to pick at a rapid pace are not typical under the DBR formulation. The latter may lead to underestimating the amplitude of a shift in condom use under the potential extrinsic influence of the Avahan intervention. For this reason, we also consider an alternative sigmoid curve, defined in the following way:

(6) $$\begin{array}{cc} U(t)= & a+\frac{b}{1+cx(t)},\\ & dx(t)=-kx(t)dt. \end{array}$$

Here the model is parameterized by its baseline $U_{0}$, its asymptote *η*, its time of inflection $\tilde{t}$ and the increase rate *k*, from which *a*,* b* and *c* can be computed:

(7) $$\left.\begin{array}{cc} & a=\eta -b,\\ b= & \left(1+\frac{1}{c}\right)\left(\eta -U_{0}\right),\\ & c=\exp(k\tilde{t}). \end{array}\right\}$$

#### 2.2.4 Stochastic growth curves

It is also possible to combine the Brownian motion and the growth curve approaches by using diffusion processes. Stochastic extensions of the DBR and deterministic sigmoid model can be considered, in which the mean behaviour remains intact although some random perturbations are introduced through a stochastic differential equation. To ensure positivity, restrict *U*(*t*) below 1 and to retain the link with the DBR curve, geometric Brownian motion can be used to replace equations (5) and (6):

(8) $$dx\left(t\right)=-kx\left(t\right)dt+\sigma x\left(t\right)dB_{t}.$$

The stochastic growth curve that is defined by equations (4) and ((8)) was also mentioned in Garcia (1983). A convenient feature for both stochastic extension of the DBR and the deterministic sigmoid models is the fact that, since

$$x\left(t\right)=\frac{1}{1-m}\left\{U{\left(t\right)}^{1-m}-\eta^{1-m}\right\},$$

and *x*(*t*) is strictly negative, the resulting condom use trajectory is maintained strictly below *η*. Given the limited data at our disposal, these models can hardly be fitted in the context of this paper. Nevertheless, they may be helpful in cases where more observations are available.

### 2.3 Priors

The parameters contained in $\theta_{t_{0}}$ and $\theta_{b}$ cannot be identified from the prevalence observations only, so we assign informative priors on them. These are summarized in Table 1 and are similar to the priors that were used in Pickles *et al*. (2010, 2013). The priors relative to general quantities as transmission probability for unprotected acts or life expectancy with HIV were based on previous literature. Cross‐sectional individual‐based surveys conducted in Mysore were used to inform the priors regarding quantities that are more sociologically and geographically specific.

**Table 1:** Table of priors for the different components of $\left\{\theta_{t_{0}},\theta_{b},\theta_{c}\right\}$

*Definition*	*Notation*	*Range of uniform priors for the district of Mysore (Pickles et al.,* 2010 *)*
*HIV transmission model parameters*		
Probability of transmission from client to sex worker per act	$p_{S}$	0.0006–0.0055
Probability of transmission from sex worker to client per act	$p_{C}$	0.0001–0.007
Condom efficacy per act	*e*	80–95%
Mean number of acts per client	*a*	1–2
Mean number of clients per high‐risk FSW	$C_{H}$	46.6–54.0 clients per month
Mean number of clients per low‐risk FSW	$C_{L}$	20–23.7 clients per month
Total number of FSWs	$N_{H}+N_{L}$	1943
Clients/FSW population ratio	$\frac{N_{C}}{N_{H}+N_{L}}$	7–19
Mean length of sexual activity as FSW	$\mu_{S}^{-1}$	45–54 months
Mean length of sexual activity as client	$\mu_{C}^{-1}$	154–191 months
Mean life expectancy after infection with HIV	$\alpha^{-1}$	87–138.5 months
Initial proportion of infected FSWs in 1985	$\frac{I_{H}+I_{L}}{N_{H}+N_{L}}$	0–5%
Initial proportion of infected clients in 1985	$\frac{I_{C}}{N_{C}}$	0–5%
*Condom trajectory priors parameters*		*Prior*
Allometric parameters (DBR)	*m*	$N\left(1,10^{6}\right)I_{]1,\infty \left[\right.}$
Growth rate (deterministic sigmoid)	*k*	$N\left(0,10^{6}\right)I_{]0,\infty \left[\right.}$
Asymptote (DBR, deterministic sigmoid)	*η*	Unif(0,1)
Initial value (all trajectory priors)	$U_{0}$	Unif(0,1)
Time of inflection (DBR, deterministic sigmoid)	$\tilde{t}$	Unif(1985,2012)
Allometric parameters, initial conditions and asymptote (DBR)	$\left(U_{0},\eta ,m\right)$	0 if $U_{0}⩾m^{1/\left(1-m\right)}\eta$
Volatility (DBM)	*σ*	Unif(0,2.1)

Although there is some information in the data regarding $\theta_{c}$, the parameters that are used to describe the condom use trajectory, their posterior will depend on the prior to a large extent. As mentioned earlier, there is very little information on the volatility parameter of the deterministic Brownian motion (DBM) formulation. Throughout this paper we used a uniform prior between 0 and 2.1. As explained in more detail in Section 3.1, the parameter of main interest in this study is the quantity Δ*U*=*U*(2009)−*U*(2003). Simulations suggest that the combination of the DBM hypothesis with a Unif(0,1) prior for $U_{0}$ (condom use on January 1st, 1985) results in a symmetric prior on Δ*U* that is centred near 0 with 2.5% and 97.5% points at ±0.6 respectively. We considered it as a reasonably vague prior for Δ*U* and evaluated the performance of the resulting model via the simulation experiments of Section 3. More diffuse priors can also be used by setting a larger value for the upper limit of the uniform prior for *σ*. The sensitivity of our analysis to these assumptions is explored in Section 4.2. Regarding the parameters of the sigmoid curves, we used vague priors that are also shown in Table 1.

### 2.4 Computational schemes for implementation

The joint posterior distribution can be calculated up to proportionality through the HIV transmission model of Section 2.1, which links the prevalence observations to the condom use trajectories, the trajectory priors of Section 2.2 and the remaining priors of Section 2.3. For the DBR and deterministic sigmoid trajectory priors, the model can be put in a non‐linear regression framework, with the non‐linear function being the solution of the ordinary differential equation and can therefore be implemented with standard software such as WinBUGS through WBDiff (Lunn, 2004). However, this approach cannot be extended to the DBM case where more involved techniques are required. Since the posterior probability density function is intractable, a data augmentation scheme can be utilized. This inference problem poses some challenges due to the high dimension of the discretized representation of $U_{0:N}$ and to the strong correlation between $U_{0:N}$ and the vector of constant parameters, *θ*. This correlation leads to poor mixing and convergence properties, when applying a Gibbs scheme to *θ* and $U_{0:N}$. The PMCMC algorithm (see Andrieu *et al*. (2010)) can be used to circumvent these difficulties by updating the two components jointly, thus reducing the problem to a small dimensional MCMC algorithm on *θ* that takes advantage of the state space structure of the model. The Metropolis–Hastings step of this algorithm is determined by the estimates of the likelihood $\hat{p}\left(y|\theta \right)$ provided by a particle smoother.

The particle smoother and the PMCMC algorithm are described respectively in algorithms 1 and 2 (Tables 2 and 3), in terms of the quantities that were introduced in the previous sections. More details about this algorithm and its practical implementation can be found in Dureau *et al*. (2013) through an application in a similar context, and in Andrieu *et al*. (2010).

**Table 2:** Algorithm 1: particle smoother algorithm

With *J* being the number of particles and *n* the number of observations, initialize $L^{0}\left(\theta \right)=1$, $W_{0}^{j}=1/J$ and sample ${\left({\tilde{U}}_{0}^{j}\right)}_{j=1,\;\ldots ,\;J}$ from $p(U_{0}|\theta )$:
*for i*=0 to *n*−1 *do*
*for j*=1 to *J do*
sample $\left(\tilde{U}{\left(t_{i},t_{i}+\delta ,\ldots ,t_{i+1}\right)}^{j}\right)$ from $p\{U\left(t_{i},t_{i}+\delta ,\ldots ,t_{i+1}\right)|\theta ,U{\left(t_{i}\right)}^{j}\}$
calculate the resulting prevalence $h{\left\{\tilde{X}\left(t_{i+1}\right)\right\}}^{j}$ by solving the ordinary differential equation (e.g. with the Euler step)
set $\alpha^{j}=p\left[y_{i+1}|h{\left\{\tilde{X}\left(t_{i+1}\right)\right\}}^{j}\right]$
*end for*
set $W_{i+1}^{j}=\alpha^{j}/\Sigma_{k=1}^{J}\alpha^{k}$, and $L^{i+1}\left(\theta \right)=L^{i}\left(\theta \right)\left(1/J\right)\Sigma \alpha^{j}$
resample ${\left(\tilde{U}{\left(t_{1},t_{1}+\delta ,\ldots ,t_{i+1}\right)}^{j},\tilde{X}{\left(t_{1},t_{1}+\delta ,\ldots ,t_{i+1}\right)}^{j}\right)}_{j=1,\;\;\ldots \;,N}$ according to $\left(W_{i+1}^{j}\right)$,
*end for*

**Table 3:** Algorithm 2: PMCMC algorithm (particle marginal Metropolis–Hastings version)

With *M* being the number of iterations, set the current *θ*‐value $\tilde{\theta }$ to an initial value, use the particle smoother according to algorithm 1 to compute $\hat{p}\left(y_{1:N}|\tilde{\theta }\right)=L\left(\tilde{\theta }\right)$ and sample $\tilde{U}{\left(t_{1},t_{1}+\delta ,\ldots ,t_{n}\right)}^{\tilde{\theta }}$ from $p\{U\left(t_{1},t_{1}+\delta ,\ldots ,t_{n}\right)|y_{1:N},\tilde{\theta }\}$
*for i*=1 to *M do*
sample ${\tilde{\theta }}^{*}$ from $Q(\tilde{\theta },\cdot )$
use the particle smoother to compute $L({\tilde{\theta }}^{*})$ and sample $\tilde{U}{\left(t_{1},t_{1}+\delta ,\ldots ,t_{n}\right)}^{{\tilde{\theta }}^{*}}$ from $\hat{p}\left\{U\left(t_{1},t_{1}+\delta ,\ldots ,t_{n}\right)|y_{1:N},{\tilde{\theta }}^{*}\right\}$
set $\tilde{\theta }={\tilde{\theta }}^{*}$ and ${\tilde{U}}^{\tilde{\theta }}\left(t_{1},t_{1}+\delta ,\ldots ,t_{n}\right)={\tilde{U}}^{{\tilde{\theta }}^{*}}\left(t_{1},t_{1}+\delta ,\ldots ,t_{n}\right)$ with probability $1\wedge L\left({\tilde{\theta }}^{*}\right)Q\left({\tilde{\theta }}^{*},\tilde{\theta }\right)/\left\{L\left(\tilde{\theta }\right)Q\left(\tilde{\theta },{\tilde{\theta }}^{*}\right)\right\}$
record $\tilde{\theta }$ and ${\tilde{U}}^{\theta }\left(t_{1},t_{1}+\delta ,\ldots ,t_{n}\right)$
*end for*

Regarding the choice of *Q*(·) in algorithm 2, we use a random‐walk Metropolis–Hastings algorithm in a transformed parameter space (log‐ or logit) to ensure boundedness constraints. Each iteration of the MCMC algorithm requires an execution of the particle smoother, which induces substantial computational cost if the importance sampling covariance matrix Σ is ill adapted. Adaptive approaches (Roberts and Rosenthal, 2009) can be used to tune Σ but they require lengthy explorations of the target space. We propose to speed up this process by pre‐exploring a proxy posterior density relying on a Gaussian approximation of the dynamic system and the extended Kalman filter methodology (Dureau *et al*., 2013). A simple bootstrap version of the particle smoother is used as it is not straightforward to consider data‐driven transition proposals given the complex observation regime of our model. Note that, given the short length of the observed time series, simpler alternatives to PMCMC sampling may perform reasonably well. For example, the resampling step can be omitted and the particle smoother output can be used to approximate $p(U_{0:N}|y_{1:N},\theta )$. However, in our application, it appears that a substantial number of additional particles are needed, thus not offering a great reduction in the computational cost when compared with PMCMC sampling. We therefore suggest the use of PMCMC sampling as a robust computational tool that still does not require a large amount of computation time in applications of this type. To meet the computational requirements of the ensemble simulations in the present paper, and to facilitate future applications of the methodology that we are proposing, the calculations were made by using the *SSM* inference package (https://github.com/JDureau/ssm). This package, targeted to time series analysis via state space models, generates executables for likelihood optimization and Bayesian inference algorithms in parallelizable C for any stochastic compartmental model expressed in a high‐level modelling grammar (see Dureau *et al*. (2014) for more information).

## 3 Evaluation methodology based on ensemble simulations

Given the limited amount of information in the data that were available (five prevalence observations, including initial conditions), it is very likely that the posterior output will be influenced substantially by the choice of *U*‐priors and their parameters. In this section we explore the performance of the proposed inferential mechanisms via simulation‐based experiments designed to mimic the behaviour of data sets that are typically encountered in the context of the motivating application. Clearly, the approach of this paper heavily relies on the HIV infection model and the results will be dependent on its specification. We therefore set up the simulation experiments under the assumption that the model of Section 2.1, parameterized according to the priors of Section 2.3, is correct. The focus is on quantities that are related with the condom use trajectories, under the different choices of Section 2.2, that can be estimated from the samples of the posterior distribution provided by the MCMC algorithms of Section 2.4. We also provide some discussion regarding the static parameters appearing in the condom use trajectory priors.

### 3.1 Parameter of interest

By fitting each of the previously introduced models, we obtain samples from the marginal posterior density $p^{M}\left\{U\left(t\right)|y\right\}$ (*M* ∈ {DBR,deterministic sigmoid, DBM}). However, our interest mainly lies in the amplitude of the shift in condom use between 2003 and April 2009, which is henceforth denoted by Δ*U*. The posterior draws of condom use trajectories can be transformed to provide samples from the posterior of this parameter of interest. The samples can then be used to form an estimator $\hat{\Delta }U^{M}$ of Δ*U* such as the posterior median of ${\hat{p}}^{M}\left(\Delta U|y\right)$. In what follows we explore the frequentist properties of this estimator, derived from each of the trajectory priors.

It may also be of interest to assess the estimating capabilities, given the limited amount of data, for the hyperparameters of the various *U*‐priors ($U_{0}$, *η*,* r*,* m*, $\tilde{t}$ and *σ*). As it turns out, there is information for some of them ($U_{0}$, *η* and $\tilde{t}$), whereas some others are difficult to estimate and are determined mostly by their prior (*r*,* m* and *σ*). Nevertheless, from a subject matter point of view, interest lies mainly on Δ*U*, whereas the remaining quantities (in the condom use priors) can be regarded as nuisance parameters. Another appealing feature of Δ*U* is that it appears in all models and therefore provides an omnibus quantity for comparison. Hence, inference properties of these parameters ($U_{0}$, *η*,* r*,* m*, $\tilde{t}$ and *σ*) are only studied indirectly through inference properties of Δ*U*.

### 3.2 Measures of performance

The performance of each estimator $\hat{\Delta }U^{M}$ in estimating Δ*U* is evaluated from the following criteria (where *L*=1000, the number of simulations):

$$\begin{array}{cc} {\text{Bias}}^{M} & =\left(1/L\right)\sum_{i}\left(\hat{\Delta }U_{i}^{M}-\Delta U_{i}\right),\\ {\text{MSE}}^{M} & =\left(1/L\right)\sum_{i}{\left(\hat{\Delta }U_{i}^{M}-\Delta U_{i}\right)}^{2},\\ {\text{Std}}^{M} & =\surd \left\{{\text{MSE}}^{M}-{\left({\text{bias}}^{M}\right)}^{2}\right\}. \end{array}$$

In addition to these quantities, we are also interested in assessing the discriminative ability of each model in detecting increases in condom use. The focus is given to increases in condom use that are at least as high as a prespecified threshold Γ, which reflects the minimum practical increase. When analysing the data a researcher may decide that condom use did increase more than Γ if the value of the estimator $\hat{\Delta }U^{M}$ is higher than a user‐specified threshold *γ*. Each decision mechanism may lead to different types of error and is therefore associated with a particular sensitivity and specificity. More specifically we can define the rate of true and false positive results in the following way:

- sensitivity (true positives rate) for *γ*, $♯(\hat{\Delta }U^{M}>\gamma ,\Delta U>\Gamma )/♯(\Delta U>\Gamma );$
- specificity (1 − false positives rate) for *γ*, $♯(\hat{\Delta }U^{M}<\gamma ,\Delta U<\Gamma )/♯(\Delta U<\Gamma )$.

We proceed by first reporting the sensitivity and specificity corresponding to the case *γ*=Γ. This corresponds to saying that Δ*U* is higher than Γ if its estimator is higher than Γ. We then use a range of *γ*s and obtain the sensitivity–specificity pairs that correspond to each of them. A lower detection threshold *γ* will increase the sensitivity of the method, but it also increases the risk for false positive results, and vice versa. These pairs are combined to form the receiver operating characteristics curve by plotting sensitivity *versus* 1 − specificity. The area under the receiver operating characteristics curve, AUC, provides an overall measure of discriminatory power as it reflects the probability of correctly classifying a randomly chosen positive instance as higher than a randomly chosen negative one (Fawcett, 2006). For example, an AUC‐value of 50% indicates no power (i.e. random choice). This detailed procedure is repeated to assess the ability to detect two different levels of increase in the use of condoms, with *B* set to 20%, 30% and 40%.

### 3.3 Simulation procedure

The performance of the estimators that are derived from the various models is measured by using a set of simulated experiments where condom use trajectories are sampled from a given growth curve model (deterministic sigmoid), and parameters from *θ* are sampled following their prior distributions. To maximize the utility of this test procedure, and to serve future application of this method in different districts targeted by the Avahan programme, only plausible and realistic condom use trajectories are considered: cases with prevalence in 2010 between 2% and 40% and with condom use shifts that occurred after 1995. Furthermore, the test trajectories have been sampled so that Δ*U* regularly spans the [0;0.9] interval. Alternatively, a second set of simulations has been generated based on stepwise condom use trajectories (constant *U* up to $\tilde{t}$, and then constant *U* after $\tilde{t}$) to explore the effect of model misspecification and to assess the sensitivity of results to the underlying model used for simulations (deterministic sigmoid).

For each of these experiments, an epidemic is simulated to provide observations $y_{i}^{s}$ replicating the observation framework applied in Mysore: three prevalence estimates among FSWs and one among clients, concentrated during the period of the intervention. From these observations, the MCMC algorithm is applied to each method *M* to sample from $p\{U^{M}\left(t\right)|y_{1:N}^{s}\}$. Then, given the posterior *U*‐samples, the estimators $\hat{\Delta }U^{M}$ can be computed and compared with their true counterparts Δ*U* through the measures of performance that were introduced in the previous Section 3.2.

## 4 Results

### 4.1 Comparison of the condom use trajectory models from ensemble simulations

This section contains the results of the simulation experiments that were conducted as described in Section 3.3. In all cases each model was fitted to each of the 1000 simulated data sets and the estimator is formed via the posterior median at the points of interest. These samples are used to assess the bias, standard deviation and mean‐squared error MSE of these estimators. Moreover, in Table 4, we examine the ability of each model to classify the simulated instances of the Δ*U*‐parameter, and we assess the risk of overstating *versus* understating. In other words, we assess the ability of model‐driven estimators to address questions such as ‘was the shift in condom use during the intervention over 0.2, 0.3 or 0.4?’, by looking at their sensitivity and specificity as well as the resulting AUC.

**Table 4:** Frequentist properties of the various estimators of the amplitude of the shift in condom use during the intervention, estimated from 1000 simulations from the deterministic empirical sigmoid model

*Statistic*	*Results for the DBR model*	*Results for the deterministic sigmoid model*	*Results for the DBM model*
Bias	−0.33	−0.31	−0.26
Error standard deviation	0.21	0.20	0.19
MSE	0.15	0.14	0.10

**Figure 2:**
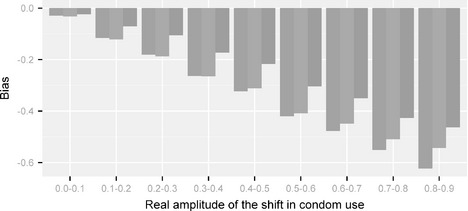
Bias of each model as a function of the true amplitude of the shift in use of condoms, estimated from 1000 simulations: 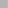, DPR model; 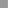, deterministic sigmoid model; 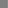, DBM model

We begin with results corresponding to cases where the data were simulated from the deterministic sigmoid model. Table 4 reports bias, standard deviation and MSE for each model‐driven estimator. It suggests that all models underestimate Δ*U*. More precisely, the DBR model tends to understate strongly the shift in amplitude, by 0.33 on average. The bias of the deterministic sigmoid model is only slightly smaller (−0.31), and optimal results are obtained with the Brownian motion model (−0.26). Similarly, in terms of MSE, the performance of the DBM model is better. Fig. 2 plots the bias against the true value of Δ*U* for each model and reveals an increasing association. Note that the ranking of the models is consistent across the entire range of Δ*U*‐values. If, for example, the true shift is between 40% and 50%, it is on average underestimated by 0.21 with the best method (DBM) and more than 0.30 points with the DBR and deterministic sigmoid methods.

The bias in estimating Δ*U* under each of the *U*‐priors can be attributed to a large extent to the prior that is implied by each formulation on Δ*U*. As mentioned in Section 2.3 the DBM approach results in a symmetric prior on Δ*U* that is centred near 0 with 2.5% and 97.5% points at ±0.6 respectively. The posterior median is therefore pulled towards 0, thus resulting in conservative estimates. The amount of shrinkage depends on the upper limit of the uniform prior on *σ*. The corresponding priors under the DBR and deterministic sigmoid formulations result in priors for Δ*U* that put more mass around zero, which heavily depends on the priors that are placed on *k* and *m*. It would perhaps be interesting in the future to consider alternative priors for *k* and *m* to reduce the bias in Δ*U* although it is not clear whether this should be the main optimization criterion. Regarding the DBM model, the uniform prior assigned on *σ* appears to work reasonably well.

**Figure 3:**
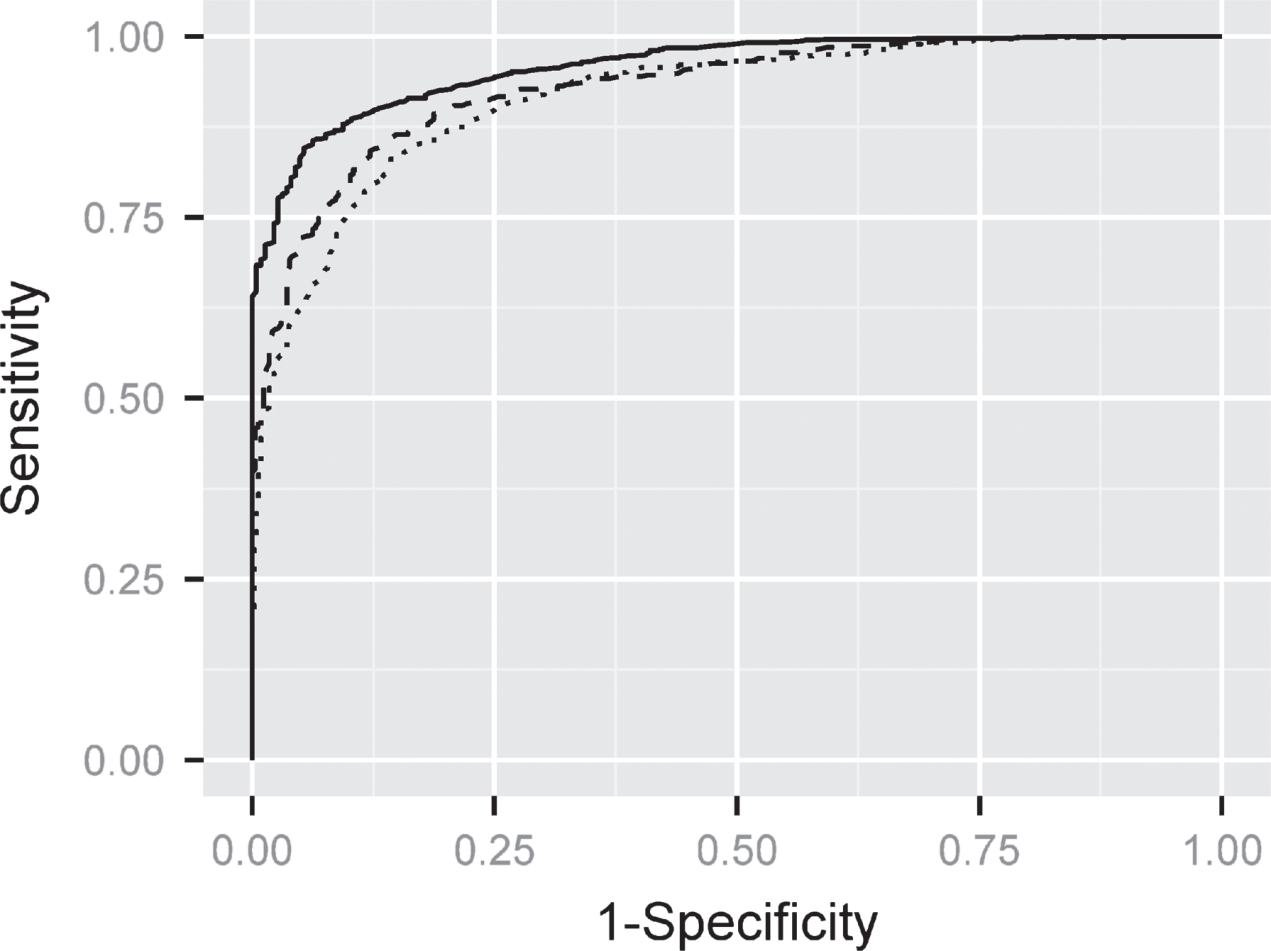
Receiver operating characteristics curve when testing for Δ*U*>0.2 ( 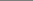), Δ*U*>0.3 (– – –) and Δ*U*>0.4 (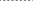), under a Brownian motion trajectory prior: the curves were estimated from 1000 simulations; very similar shapes are obtained for the alternative trajectory priors

Table 5 suggests that all estimators based on the median of the posterior density of $p(\Delta U|y_{1:N})$ have good distinguishing power: the AUC is between 0.87 and 0.93 in all cases. In line with the results of Table 5, the estimates provided by the DBM model achieve better sensitivity (53%, 29% and 14%) than the other models (between 0% and 29%). The strong negative bias that is observed in Table 5 is also reflected by an optimal 100% specificity of the estimators. The performance, in terms of sensitivity, decreases as the level of Δ*U* increases. Fig. 3 also depicts the ROC curve for the DBM model, obtained as described in Section 3.3.

**Table 5:** General distinctive power (AUC) of the median estimator of the shift, and specific sensitivity and specificity when answering the question is the shift in condom use during the intervention stronger than 0.2, than 0.3 and than 0.4?^a^

*Question*	*Parameter*	*Results for the DBR model*	*Results for the deterministic sigmoid model*	*Results for the DBM model*
Δ*U*>0.2?	AUC	0.93	0.93	0.92
	Sensitivity	26%	29%	53%
	Specificity	100%	100%	100%
Δ*U*>0.3?	AUC	0.92	0.92	0.89
	Sensitivity	5%	14%	29%
	Specificity	100%	100%	100%
Δ*U*>0.4?	AUC	0.90	0.89	0.87
	Sensitivity	0%	7%	14%
	Specificity	100%	100%	100%

a These quantities were estimated from 1000 simulations from the deterministic empirical sigmoid model.

In an additional simulation experiment we examine the frequentist properties of the models in the case where underlying condom use trajectories were generated from a step function. As shown in Table 6, a very similar picture is obtained with the DBM model having the smallest bias and MSE, being slightly better than the DBR model.

**Table 6:** Frequentist properties of the various estimators of the amplitude of the shift in condom use during the intervention, estimated from 1000 simulations from stepwise condom use trajectories (constant *U* until $\tilde{t}$, and constant *U* after $\tilde{t}$)

*Statistic*	*Results for the DBR model*	*Results for the deterministic sigmoid model*	*Results for the DBM model*
Bias	−0.35	−0.33	−0.26
Error standard deviation	0.21	0.20	0.18
MSE	0.16	0.15	0.10

Overall, the results that are presented in Tables 4, 5, 6 provide an informative qualitative assessment of the ability of the various models to capture Δ*U* from limited prevalence data on an important and diverse set of likely scenarios (1000 experiments with true Δ*U* spanning between 0 and 0.9). First of all, MSE‐ and AUC‐figures suggest that, although the number of prevalence observations is low and some elements of the transmission process are uncertain, it is still possible to extract information on our time varying parameter and to provide estimates of Δ*U* during the intervention. Furthermore, there seems to be a possibility of controllig the risk of overstating these quantities by analysing the outputs of the three models, that offer different levels of compromise between sensitivity and specificity. Thus, although the procedure may fail to identify some shifts in use of condoms, there seems to be some reassurance that once a shift has been detected it will most probably be true.

Finally, note that the two models with the higher overall performance, the DBM and deterministic sigmoid model, are quite different in nature: the deterministic sigmoid trajectories are smooth, whereas under the Brownian motion prior they are non‐differentiable. Hence, the choice between the two models can also be based on prior beliefs of the researcher regarding the smoothness of the condom use trajectories.

### 4.2 Application: what can we infer on the trajectory of condom use in Mysore?

**Figure 4:**
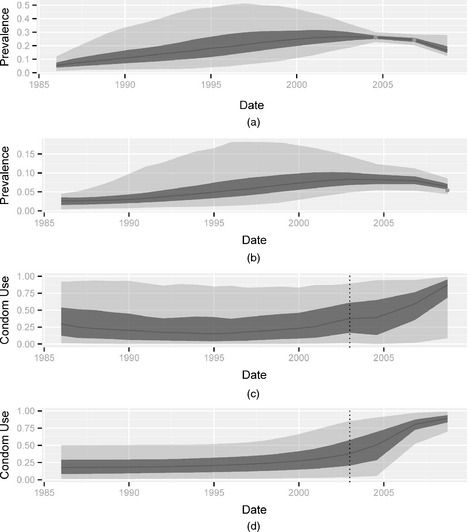
Estimates obtained for the Mysore district: (a) reconstructed prevalence trajectory among FSWs when condom use is modelled with Brownian motion; (b) reconstructed prevalence trajectory among clients when condom use is modelled with Brownian motion; (c) reconstructed condom use trajectory when modelled with Brownian motion; (d) reconstructed condom use trajectory when modelled with the deterministic sigmoid model

Mysore is one of the districts that were targeted by the Avahan intervention, and this programme was the first HIV prevention intervention in this region. Four HIV prevalence estimates have been obtained between 2003 and 2009: three among FSWs, and one among clients. Results from the inference procedure using a Brownian motion model are shown in Fig. 4, suggesting a strong effect of the intervention. The purpose of this paper was to determine what level of increase of condom use between 2003 and 2009 can be inferred while controlling the risk of overstating it. As was shown in Section 4.1, deterministic sigmoid models could provide a good alternative to the DBM formulation. Hence, we also present here results obtained with this model for the Mysore data set (see Fig. 4). Note that the posterior uncertainty around the condom use trajectory is lower in the case of the deterministic sigmoid model compared with the DBM model. This is in line with our expectations given the fact that this prior imposes a particular growth structure and therefore introduces additional information. Loosely speaking this may be viewed as a bias–variance trade‐off. In general, the choice of each *U*‐formulation is likely to affect the inference procedure and therefore it is useful to explore its implications, e.g. via the simulation experiments of the previous section. Table 7 shows the estimates of Δ*U* for each of the three models presented. The results indicate a positive increase in all cases. In particular, the posterior medians for the DBM and deterministic sigmoid models are 0.54 and 0.49 whereas the 95% credible intervals are [0.06;0.95] and [0.09;0.97] respectively.

**Table 7:** Estimates of the change in condom use in Mysore between 2003 and 2009

*Model*	*Posterior mean*	*Posterior median*	95% *credible interval*
DBR	0.30	0.29	[0.09;0.54]
Deterministic sigmoid	0.48	0.49	[0.09;0.87]
DBM	0.52	0.54	[0.06;0.95]

A stronger conclusion regarding a lower bound for the condom use shift between 2003 and 2009 can be made by comparing the posteriors medians with the results of Table 7. If the underlying set of simulations is to be considered realistic, an argument in favour of an increase in the use of condoms being at least 0.4 can be made. Since the posterior medians are more than 0.4 under both DBM and deterministic sigmoid models (0.54 and 0.49 respectively), Table 7 suggests that a statement for Δ*U*>0.4 will be correct with probability given by the specificity of each model (100% in both cases). Although more informative than the credible intervals that are obtained directly from the posterior densities (over 0.06), these numbers are heavily dependent on the assumption that the simulations of Section 3 provided an adequate approximation of the reality and should therefore be treated with caution. Fig. 4 and Table 7 show that the results that are obtained from the deterministic sigmoid and Brownian motion models strongly coincide: they suggest that condom use was stable over the period 1985–2003, remaining below 0.5, sharply increased between 2003 and 2007, and kept growing until 2009.

Finally, Table 8 summarizes the results for sensitivity analysis regarding the choice of priors for the parameters *σ* (DBM model) and $\tilde{t}$ (DBR and deterministic sigmoid models). As we can see from the results of Table 8, the posterior summaries are not altered substantially for a number of prior formulations, suggesting a reasonable amount of robustness to their specification.

**Table 8:** Sensitivity analysis: effect on the prior on *σ* and $\tilde{t}$ on the estimated changes in condom use

*Model*	*Parameter*	*Prior density*	*Posterior mean*	*Posterior median*	95% *credible interval*
DBM	*σ*	Unif(0,2.7)	0.578	0.613	[0.048;0.975]
		Unif(0,2.4)	0.509	0.525	[0.029;0.964]
		Unif(0,2.1)	0.516	0.529	[0.045;0.961]
		Unif(0,1.8)	0.463	0.448	[0.059;0.918]
		Unif(0,1.5)	0.465	0.461	[0.035;0.874]
DBR	$\tilde{t}$	Unif(1985,2012)	0.309	0.299	[0.084;0.589]
		Unif(1985,2009)	0.307	0.294	[0.081;0.593]
		Unif(1988,2012)	0.309	0.299	[0.084;0.589]
		Unif(1982,2012)	0.309	0.299	[0.085;0.589]
Deterministic	$\tilde{t}$	Unif(1985,2012)	0.471	0.487	[0.054;0.903]
		Unif(1985,2009)	0.462	0.474	[0.057;0.903]
		Unif(1988,2012)	0.472	0.487	[0.054;0.903]
		Unif(1982,2012)	0.472	0.487	[0.054;0.903]

In summary, the results of this section support the findings of other independent modelling (Boily *et al*., 2008; Pickles *et al*., 2010) and empirical epidemiological studies (Lowndes *et al*., 2010; Deering *et al*., 2008; Bradley *et al*., 2010) that suggested that the use of condoms has increased substantially in Mysore following the start of the Avahan programme. However, all these other studies have been based on approaches that strongly rely on self‐reported condom use data which can be biased. Our method here has the advantage of not imposing any strong prior on the presence and timing of consequences of the intervention. Together, this new and previous analysis support recent analysis of the effect of the Avahan programme in reducing HIV infection among FSWs and clients in Mysore (Pickles *et al*., 2010).

## 5 Discussion

In this paper, we presented a Bayesian approach to draw conclusions regarding the evolution of time varying behavioural parameters in the context of HIV such as the use of condoms among FSWs. Inference can be based on prevalence estimates and a substantial amount of information from additional sources can be incorporated via prior distributions. To describe the behaviour of condom use trajectories we introduced three different formulations based on Brownian motion and growth curves such as the generalized Bertalanffy–Richards and empirical sigmoid models. To our knowledge, these formulations are new in this context. The computational framework presented allows estimation of condom use trajectories as well as functionals thereof, using advanced MCMC methods and following ideas of Dureau *et al*. (2013), Cazelles and Chau (1997) and Rasmussen *et al*. (2011). Nevertheless, in comparison with these approaches, the problem of evaluating the Avahan intervention by estimating its effect on the use of condoms from prevalence estimates is of additional difficulty due to the limited amount of information; the application to Mysore district was based on three observations of prevalence among FSWs and one among clients, plus a hypothesis on the initial value of prevalence in 1985. Various simulation experiments were conducted to assess the validity of the procedure, examining the frequentist properties of the underlying estimators and the ability of the model to avoid overestimation via conducting receiver operating characteristics analysis. The evidence from the simulation experiments is encouraging, suggesting that the approach can be used in this context for making conservative estimates of changes in the use of condoms with both the Brownian motion and the deterministic sigmoid trajectory priors. However, the overall performance is bound to depend on the deterministic HIV infection model which was parameterized on the basis of a substantial amount of prior information, as in Pickles *et al*. (2010), as well as on assumptions such as the very low HIV prevalence in 1985. Most of the prior information utilized in this study was obtained from additional data sources (individual biobehavioural surveys). In the presence of all these sources of data, it would be interesting to consider and contrast a joint inferential scheme through an evidence synthesis framework in the spirit of Goubar *et al*. (2008) and Presanis *et al*. (2011).

Although the representation of the natural history of HIV transmission in this paper is simpler in behavioural terms in comparison with the model that was presented in Pickles *et al*. (2010), the model adopted retains the heterogeneity in FSW behaviour and it is enriched by exploring the condom use trajectories space rather than working with predetermined scenarios. Nevertheless, there are reasons for a potential overestimation of the shift amplitude in this simpler model as coinfections with other sexually transmitted diseases were ignored (although higher transmission probabilities per unprotected act were allowed to compensate for them), and no acute phase was considered. However, diffusion‐driven models aim at capturing and compensating for structural misspecifications while capturing the main dynamics of the system and have been shown here to provide conservative estimates. Overall it may be viewed as a different and complementary choice in the trade‐off between richness and tractability of the model compared with Pickles *et al*. (2010, 2010. Lastly, this approach relies on the hypothesis that changes in transmission probabilities are solely related to changes in the use of condoms, ignoring for example potential changes in the frequency of commercial sex partnerships. This choice can be motivated by the strong focus of the Avahan intervention on prevention measures and the relative stability in the frequency of commercial sex that are exhibited by the series of cross‐sectional biobehavioural surveys that were conducted during the period of the intervention.

From a wider perspective, the present paper provides a generic methodology for applications of diffusion processes coupled to mechanistic epidemiological models in an evaluation monitoring framework. Naturally, this methodology can be used to study the effect of the Avahan intervention in the other regions of India that were targeted by the programme. In a different context, the approach that was presented in this paper has been applied in the context of the 2014 Ebola virus outbreak to monitor in realtime the effect of the interventions conducted in Liberia, Sierra Leone and Guinea (Camacho *et al*., 2014). Lastly, the applicability of the methodology presented and more generally of the use of diffusion models to capture the time varying drivers of epidemics will be facilitated by the development of a generic inference toolbox for compartmental models (Dureau *et al*., 2014), motivated in part by the computational challenges that were encountered in the present paper. Examples of such applications are the study of the natural history of dengue or foot‐and‐mouth disease.
